# Evaluation of resection of the gastroesophageal junction and jejunal interposition (Merendino procedure) as a rescue procedure in patients with a failed redo antireflux procedure. A single-center experience

**DOI:** 10.1186/s12893-018-0401-8

**Published:** 2018-08-30

**Authors:** Apostolos Analatos, Mats Lindblad, Ioannis Rouvelas, Peter Elbe, Lars Lundell, Magnus Nilsson, Andrianos Tsekrekos, Jon A. Tsai

**Affiliations:** 1 Centre for Digestive Diseases, Karolinska University Hospital and Division of Surgery, Department of Clinical Intervention and Technology (CLINTEC), Karolinska Institutet, Stockhom, Sweden; 2 Department of Surgery, Nyköping Hospital, Nyköping, Sweden; 3 Centre for Clinical Research Sörmland, Uppsala University, Uppsala, Sweden

**Keywords:** Gastroesophageal reflux, Reoperation, Quality of life, Jejunal interposition, Merendino procedure

## Abstract

**Background:**

Primary antireflux surgery has high success rates but 5 to 20% of patients undergoing antireflux operations can experience recurrent reflux and dysphagia, requiring reoperation. Different surgical approaches after failed fundoplication have been described in the literature. The aim of this study was to evaluate resection of the gastroesophageal junction with jejunal interposition (Merendino procedure) as a rescue procedure after failed fundoplication.

**Methods:**

All patients who underwent a Merendino procedure at the Karolinska University Hospital between 2004 and 2012 after a failed antireflux fundoplication were identified. Data regarding previous surgical history, preoperative workup, postoperative complications, subsequent investigations and re-interventions were collected retrospectively. The follow-up also included questionnaires regarding quality of life, gastrointestinal function and the dumping syndrome.

**Results:**

Twelve patients had a Merendino reconstruction. Ten patients had undergone at least two previous fundoplications, of which one patient had four such procedures. The main indication for surgery was epigastric and radiating back pain, with or without dysphagia. Postoperative complications occurred in 8/12 patients (67%). During a median follow-up of 35 months (range 20–61), four (25%) patients had an additional redo procedure with conversion to a Roux-en-Y esophagojejunostomy within 12 months, mainly due to obstructive symptoms that could not be managed conservatively or with endoscopic techniques. Questionnaires scores were generally poor in all dimensions.

**Conclusions:**

In our experience, the Merendino procedure seems to be an unsuitable surgical option for patients who require an alternative surgical reconstruction due to a failed fundoplication. However, the small number of patients included in this study as well as the small number of participants who completed the postoperative workout limits this study.

## Background

Fundoplication in patients with chronic gastroesophageal reflux disease (GERD) is generally followed by excellent short- and long-term results [1–5]. Failures are, however, unavoidable and present with recurrent reflux symptoms, postprandial pain, dysphagia and delayed gastric emptying [6]. A variety of different mechanisms may cause these symptoms such as recurrent hiatal hernia, dislocation of the fundoplication, vagus nerve damage and/or other morphological abnormalities in the hiatus [7, 8]. The problem with many of these symptoms (with the exception of acid reflux), appearing after a defective surgical repair, is that the result of conservative treatment is usually poor which consequently leads to the need for another fundoplication, with the aim to achieve a durable repair [9, 10]. With very few exceptions, the reported postoperative morbidity is significantly higher after redo surgery, which is due to the complexity of the anatomical region, postoperative scarring and deformation [11–15]. If severe symptoms recur after a second or third fundoplication remedial surgical interventions have been advocated including total gastrectomy or gastric bypass [16, 17]. The outcome of these procedures has often been reported as good to excellent but a substantial publication bias in favour of respective surgical intervention may be present. In addition, a number of potentially relevant factors have to be taken into consideration. Maintenance of the gastrointestinal continuity with preservation of the duodenal and proximal jejunum contact with ingested food particles and prevention of reflux into the esophagus are critically important factors as well as the role of the gastric reservoir, which may be relevant for preventing post-gastrectomy symptoms. An additional symptom component, which may be prevalent, is chronic epigastric pain radiating to the back with or without exacerbation after ingestion of food. The mechanisms behind these complaints are poorly understood, but the need for surgical clearance and resection has been advocated [18].

Resection of the gastroesophageal junction with interposition of a jejunal segment between the distal esophagus and the remnant stomach (Merendino procedure) has been used in various clinical situations, mainly in patients with peptic strictures during the pre-proton pump inhibitor era and more recently for early Barrett cancer, where encouraging results have been reported [19–22]. Proximal gastrectomy with jejunal interposition for non-advanced proximal gastric cancer, which is similar to the Merendino procedure, is performed mainly in the Far East and has been shown to induce fewer post gastrectomy symptoms as compared to total gastrectomy with Roux-en-Y reconstruction, without adding more postoperative complications [23, 24]. Therefore, the Merendino procedure may have the potential to offer significant advantages in challenging post-fundoplication situations. Hereby, we report a single institution’s experience of this reconstruction method in patients with a history of failed redo antireflux surgery.

## Methods

### Identification and inclusion of patients

All patients who underwent a Merendino procedure between 2004 and 2012 at the Karolinska University Hospital, for other causes than cancer, were identified via the computerised patient records and registries for surgical procedures (Take Care, Orbit, HOPA). Patient records were reviewed and data regarding previous surgical history, preoperative workup, postoperative complications, subsequent investigations and re-interventions were collected. In 2013 all study patients, who still had an intact Merendino reconstruction were contacted and asked to participate in a follow-up with questionnaire regarding quality of life, gastrointestinal symptoms and the dumping syndrome. During the course of the management of all GERD patients, barium swallow, esophageal manometry and ambulatory 24-h pH measurement had previously been performed. The Stockholm Local Ethics Committee had approved this study and all patients who participated in the study gave their written informed consent.

### Surgical procedure

**Fig. 1:**
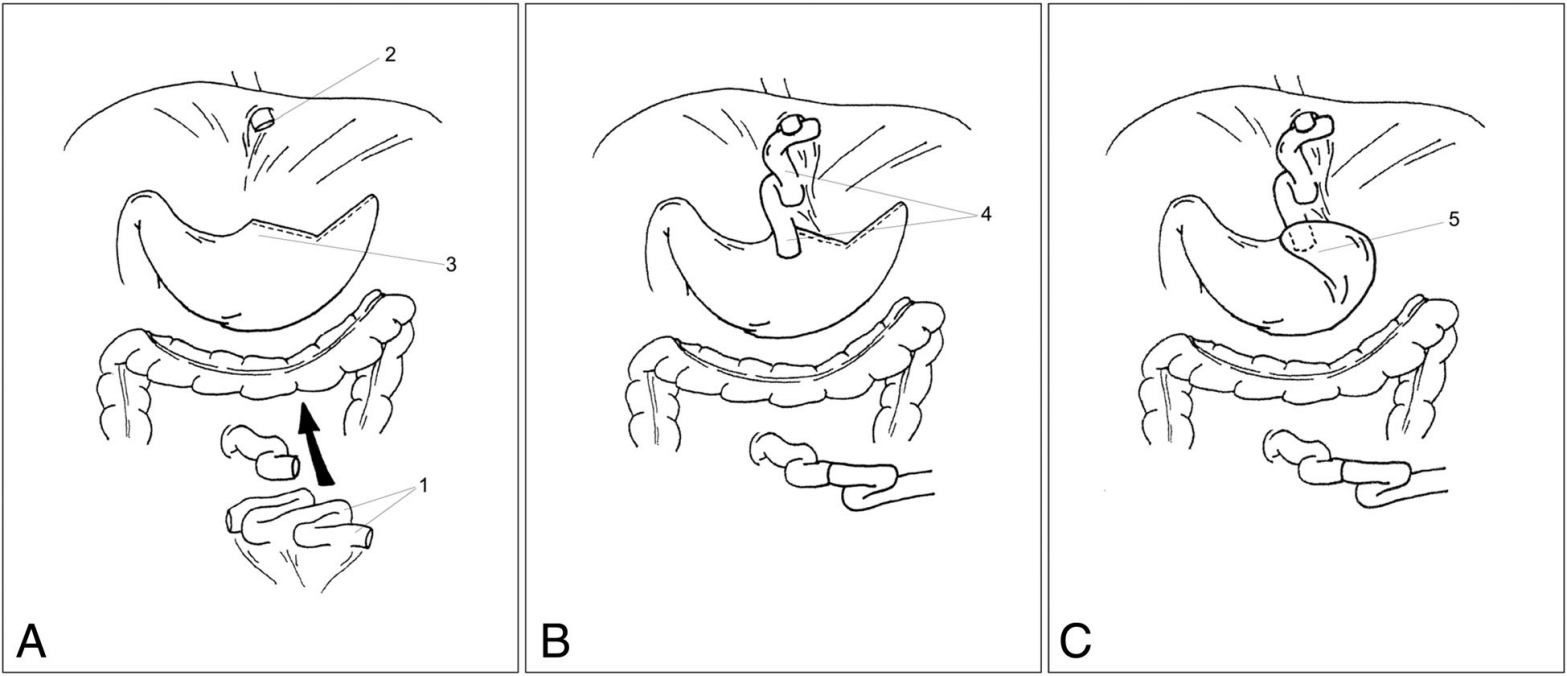
The surgical procedure of merendino. **a**: After division of the distal esophagus and the fundus of the stomach, a 30 cm long pedunculated jejunal segment is prepared [1] and drawn upwards through the transverse mesocolon (arrow). **b**: The jejunal segment is hand-sutured end to side to the distal esofagus [2] and end to side to the minor curvature of the stomach [3], in an isoperistaltic fashion [4]. **c**: A partial anterior fundoplication is created by use of the most oral portion of the major curvature of the remaining stomach [5]

Resection of the distal esophagus, cardia and proximal stomach with jejunal interposition was done via an upper midline laparotomy. A wide phrenotomy was usually performed to expose the lower posterior mediastinum and allow a safe dissection of the distal esophagus proximal to the area of previous fundoplication, which typically contained abundant scar tissue and in one case a large epiphrenic diverticulum. The dissection of the proximal stomach was also performed beyond the area of the fundoplication. After division of the distal esophagus and the fundus of the stomach, a 30 cm pedunculated, isoperistaltic jejunal segment was prepared and hand-sutured end to side to the distal esophagus and end to side to the minor curvature side of the body of the stomach with absorbable interrupted sutures (Fig. 1). In 4 patients a pyloroplasty was also added. In order to protect the jejunum from refluxed gastric content, either a posterior or anterior fundoplication was created by use of the most oral portion of the major curvature side of the remaining stomach. All patients also had a feeding jejunostomy tube placed for temporary enteral nutrition.

### QoL and symptom questionnaires

Patients eligible for the postoperative questionnaires received information about the study by telephone and then received three questionnaires by mail – the Quality Of Life in Reflux And Dyspepsia (QOLRAD), the Gastrointestinal Symptom Rating Scale (GSRS) and the Dumping Symptom Rating Scale (DSRS) instruments [25–27]. The QOLRAD questionnaire includes 25 questions divided into 5 dimensions: emotional distress, sleep disturbance, vitality, food/drink problems and physical/social function and is rated on a seven-point graded Likert scale, with low values indicating a more severe impact on daily functioning. Symptoms related to general gastrointestinal symptoms were assessed by GSRS, which is a disease specific instrument of 15 items combined in 5 different symptom-categories such as reflux, abdominal pain, indigestion, diarrhoea and constipation. The GSRS also has a seven-point graded Likert-type scale in which each symptom-cluster can take a score from 1 to 7, where 1 represents absence of symptoms and 7 very intense symptoms. The dumping syndrome (DS) was scored by the newly developed DSRS questionnaire. This includes questions regarding 11 typical symptoms (fatigue, palpitations, sweating/flushing, cold sweats/paleness, need to lie down, diarrhoea, nausea/vomiting, stomach cramp, fainting esteem, pain and vomiting) associated with the DS of which 9 items represents symptoms that occur after meals. The severity of each symptom during the past week is graded on a seven-point Likert-scale where 1 represents “no trouble at all” and 7 “very severe problems”. The frequency of 9 of the DS symptoms in the last 2 weeks is measured on a six-point Likert-scale where 1 represents “no trouble at all” and 6 “several times a day”. The mean of the severity items is the severity score and the mean of the frequency items is the frequency score and the total DSRS score is calculated by multiplying the severity score with the frequency score.

### Manometry of the esophagus and the interposed jejunal segment

Esophageal manometry investigations of the esophagus and the interposed jejunal segment were performed at the Karolinska University Hospital. A solid state High Resolution Manometry (HRM) assembly with 36 solid-state sensors spaced at 1-cm intervals was used (Sierra Scientific Instruments Inc., Los Angeles, CA) [28]. Each sensor is circumferentially sensitive and zeroed to atmospheric pressure. The HRM assembly was passed transnasally and positioned to record from the native esophagus, through the interposed jejunal segment into the gastric reservoir. The manometric protocol included a 5-min period to assess basal pressures and ten 10-mL water swallows. Manometric data were analyzed using both ManoView analysis software (Sierra Scientific Instruments Inc., Los Angeles, CA) and custom programs written in Matlab (The MathWorks Inc., Natick, MA).

### Statistics

All the collected data were stored in a database and analyzed using SPSS statistical software. Data are presented as median and range. Body weight before surgery and at follow-up was compared using the Mann-Whitney test. A *p*-value less than 0.05 was considered statistically significant.

## Results

### Previous surgical history, indications for Merendino procedure and preoperative work-up

Twelve patients (mean age of 55, range 42–66 years) having a Merendino procedure due to failed previous antireflux surgery were identified during the study period. Males dominated (10:2) and all but ten patients had a history of two or more surgical interventions and one patient had been operated on four times. The most common indication for the Merendino procedure was chronic or intermittent pain with the majority taking daily morphine derivatives to control pain (Table 1). All patients were investigated with a CT-scan and upper GI endoscopy, which revealed miscellaneous abnormalities and some of the patients were also investigated with 24 h pH measurement, esophageal manometry or esophageal barium swallow (Table 2).

**Table 1:** Demographic data, indication for the first fundoplication and the subsequent Merendino procedure

patient	Age	sex	number of previous fundoplications	Indication for the first fundoplication	indication for merendino
1	55	M	4	Gastroesophageal reflux	Dysphagia and pain
2	61	M	2	Gastroesophageal reflux	Dysphagia and pain
3	42	M	2	Gastroesophageal reflux	Recurrent Reflux
4	65	F	2	Gastroesophageal reflux	Recurrent reflux and dysphagia
5	46	M	1	Gastroesophageal reflux	Dysphagia and pain
6	61	M	2	Paraesophageal hernia	Dysphagia and pain
7	66	M	2	Gastroesophageal reflux	Dysphagia and pain
8	42	M	2	Gastroesophageal reflux	Dysphagia and pain
9	57	M	1	Paraesophageal hernia	Recurrent reflux and dysphagia
10	49	M	2	Gastroesophageal reflux	Dysphagia and pain
11	51	M	3	Paraesophageal hernia	Dysphagia and pain
12	56	F	3	Paraesophageal hernia	Dysphagia and pain

**Table 2:** Preoperative workup for patients who underwent a Merendino procedure

patient	Ph monitoring	manometry	gastroscopy	CT	esophageal Barium swallow
1	ND	ND	Paraesophageal hernia	Paraesophageal hernia	ND
2	ND	ND	Normal	Normal	Esophageal dyskinesia
3	Pathological reflux	Dyschinesia	Sliding hernia	Paraesophageal hernia	ND
4	Pathological reflux	Normal	Normal	Normal	Paraesophageal hernia
5	Normal	Normal	Normal	Paraesophageal hernia	ND
6	ND	ND	ND	Normal	Esophageal dyskinesia
7	ND	Dyschinesia	ND	Other	ND
8	ND	ND	Esophageal diverticulum	Esophageal diverticulum	Esophageal diverticulum
9	Normal	ND	Normal	Paraesophageal hernia	Paraesophageal hernia
10	ND	ND	ND	Other	ND
11	ND	ND	ND	Paraesophageal hernia	Paraesophageal hernia
12	ND	Dyschinesia	ND	Paraesophageal hernia	ND

*ND* = No Data available

### Intra-operative data, postoperative complications, length of stay, follow-up time, weight loss

The median operating time was 338 (range 197–418) minutes and the median perioperative bleeding was 500 (range 250–2000) ml. Postoperative complications were common and surgical complications occurred in 7 patients (Table 3). The postoperative course was graded according to Clavien Dindo grading system for the classification of surgical complications [29, 30]. Five patients had grade 0-I, two patients grade II, three patients grade IIIb, one patient grade IVa and one patient grade IVb. Three patients needed an acute re-operation, two because of bleeding and one because of rupture of the hiatal closure with herniation of the left colon and small bowel into the left thorax. Two patients had an anastomotic leak in the esophagojejunal anastomosis that was successfully treated with covered self-expanding metal stents. Five patients developed pneumonia and four severe septicaemia, which were successfully treated with i.v. antibiotics. Three patients developed pleural effusion that was treated conservatively (*n* = 1) or with thoracocentesis (*n* = 2). The median postoperative hospital stay was 11 days (range 6–70) and follow-up ranged from 20 to 61 months (median 35). All patients had lost weight at follow-up compared to preoperative levels, 63.5 (46–90) vs. 72.5 (50–100) kg, *p* < 0.001.

**Table 3:** Postoperative complications and Clavien-Dindo grade

Patient	Complication	Treatment	Clavien-Dindo grade
1	Pneumonia, pleural effusion	antibiotics, thoracocentesis	II
2	None		0
3	Artial fibrilation, septicaemia, bleeding, wound rupture, abdominal abscess, pneumonia, anastomotic leak	reoperation × 3, stent, drainage and antibiotics	IIIb
4	bleeding, septicaemia, pulmonary septic embolism	Reoperation, antibiotics	IIIb
5	pneumonia, small bowel paralysis	Antibiotics	II
6	None		0
7	anastomotic leak, pneumonia, septicaemia, respiratory failure, mediastinitis, pleural effusion	Reoperation, stent, antibiotics, ventilator support,	IVa
8	bleeding, pneumonia	Reoperation, antibiotics	IIIb
9	none		0
10	none		0
11	none		0
12	respiratory failure, pleural effusion, septicaemia	Ventilator support, thoracocentesis, antibiotics	IVb

### Endoscopic reinterventions and redo surgery

Dysphagia was common and resulted in re-endoscopy in five patients (Table 4). In one patient a clear stricture was found in the proximal anastomosis 26 months after surgery, which was dilated. A re-endoscopy was combined with endoscopic dilatation of the esophagojejunostomy in three of these patients and in the jejunogastrostomy in two of the patients even though the macroscopic picture of the respective anastomotic areas revealed a patent lumen and no clear stricture. Symptoms suggestive of gastric outlet obstruction, possibly due to vagal damage, occurred in three patients who subsequently underwent a dilatation of the pylorus to 35 mm using a pneumatic balloon with a temporary positive effect. Five patients developed severe symptoms including dysphagia, vomiting and weight loss postoperatively, which occurred after an essentially uneventful postoperative recovery period. These complaints could not be managed conservatively or via endoscopic interventions and therefore another reoperation was considered indicated. Four patients underwent a conversion to a RNY esophagojejunostomy without resection of the distal stomach and one patient was re-operated with resection of the blind segment in the esophagojejunal anastomosis due to a presumed pseudodiverticulum formation. However, symptoms persisted in this patient, who finally received a percutaneous endoscopic gastrostomy for nutrition. These patients have not undergone any further surgical or endoscopic interventions of the upper gastrointestinal tract.

**Table 4:** Weight before surgery and at follow-up, postoperative endoscopic interventions, redo-surgery and follow-up time among patients who underwent a Merendino reconstruction

Patient	Weight preoperatively (kg)	Weight at follow up (kg)	Endoscopic Interventions	Reoperations	Follow up (months)
1	100	90	Proximal anastomosis ×3	RNY with J-pouch	65
2	100	83	Proximal anastomosis ×2 and pylorus ×2	RNY	46
3	104	85			29
4	90	68			20
5	50	46	Proximal anastomosis ×1, distal anastomosis ×1	resection of the blind segment in the esophagojejunal anastomosis	36
6	64	52			48
7	82	65			35
8	72	73			61
9	53	50			31
10	66	62	Distal anastomosis ×1 and pylorus ×2	RNY with J-pouch	44
11	73	61		RNY	33
12	72	49	Pylorus ×2		30
	72,5 (49,6–100)^a^	63,5 (46–89,9)^a^*			35(20–61) ^a^

^a^Median (range), * = *p* < 0.001 compared to preoperative weight

### QoL and symptom score at long-term follow up

QOLRAD, GSRS and DSRS were obtained from 6 patients who still had a Merendino reconstruction and are presented in Table 5. Scores in all dimensions were generally poor. Physical/social functioning and vitality were the dimensions with the worst scores in QOLRAD, while abdominal pain and indigestion were the most intense symptoms in GSRS. Fatigue, nausea and vomiting/stomach cramps were the dimensions with the worst scores in DSRS both regarding severity and frequency. All patients except one were on an oral diet at follow-up. Four of the patients used proton-pump inhibitors (PPI) on an everyday basis due to reflux- like symptoms and one patient was treated on demand.

**Table 5:** QOLRAD, GSRS and DSRS scores from 6 patients with Merendino reconstruction presented as median (range)

QOLRAD	median (range)	GSRS	median (range)	DSRS	median (range)
Emotional distress	2.9 (2.5–5.3)	Reflux	3.8 (1.7–5.0)	Severity score	4.0 (3.0–5.4)
Sleep disturbance	2.7 (2.2–3.8)	Abdominal Pain	5.2 (3.0–5.3)	Frequency score	3.9 (3.0–5.3)
Food/drink problems	2.8 (1.7–4.2)	Indigestion	4.3 (3.0–4.3)	Total Score	15.3 (9.7–28.8)
Physical/social functioning	3.1 (1.8–5.0)	Diarrhoea	3.3 (1.0–5.0)		
Vitality	1.7 (1.0–3.3)	Constipation	3.7 (1.3–5.0)		

In QOLRAD a score of 1 represents the lowest possible quality of life and 7 the highest. In GSRS 1 corresponds to absence of symptoms and 7 very intense symptoms. In the DSRS severity score each dumping symptom during the past week is graded from “no trouble at all” [1] to “very severe problems” [7]. In the DSRS frequency score the frequency of the symptoms during the last 2 weeks is graded from “no trouble at all” [1] to “several times a day” [6]. The mean of all severity items is the severity score and the mean of all frequency items is the frequency score. Each severity item is multiplied by the respective frequency item to a DSRS total score (maximum score 42)

### Investigations of the esophagus and the interposed jejunal segment at follow-up

Four of the patients with Merendino reconstruction had a HRM after surgery. Two were examined due to weight loss and dysphagia and regurgitation and 2 patients as a part of the follow-up of this study. HRM was normal in three patients (peristalsis and motility in esophagus and jejunal segment) whereas in one patient the manometry revealed a totally aperistaltic jejunal segment. This patient and one of those with a normal HRM underwent conversion to Roux-en-Y esophagojejunostomy.

Six of the patients were investigated postoperatively with esophageal barium swallow, due to dysphagia and regurgitation. In 3 patients there were signs of stenosis in the esophagojejunal anastomosis area and in 5 patients there was a significant prolongation of barium passage through the interposed small intestinal segment. In 4 patients there were signs of reflux into the esophagus from the contrast dye accumulated in the interposed segment.

## Discussion

To our knowledge the series of cases presented herein is the first describing the results of a Merendino reconstruction, i.e. jejunal interposition between the esophagus and stomach, in patients with previously failed redo-fundoplication. We found that the rate of postoperative complications was high and at least in the same range as in previous case series of RNY reconstruction in similar patients [16, 17]. In addition, re-interventions for postoperative symptoms were frequent. Alleged anastomotic narrowing were managed by endoscopic dilatations and some patients also presented with gastric outlet obstruction symptoms suggesting pyloric dysfunction as a result of vagal damage, which may occur after repeated surgery in the hiatus area. Dilatation of the pylorus was performed in these cases, but symptoms typically persisted. As many as 4 of the 12 patients, developed postoperative symptoms of intolerable severity and therefore a redo operation and conversion to RNY esophagojejunostomy was performed. Among the patients where a questionnaire-based follow-up was available, QoL was generally poor and gastrointestinal symptoms were common. Even dumping symptoms, the risk of which, from a theoretical point, should be minimized, given the preservation of the duodenal passage and maintenance of the gastric reservoir, were also frequently reported. Taken together these data, even though the number of patients is limited, suggests that the Merendino procedure is unsuitable for the complex group of patients where redo surgery after repeat fundoplication is considered. This is important to bear in mind, especially since an additional aspect in favour of resection of the gastroesophageal junction area, was the severe pain hypothetically related to the extensive scarring of the hiatal region. Redo surgery after fundoplication using other approaches than yet another fundoplication are rarely performed and there is no evidence supporting the use of any specific reconstruction method over the other. RNY reconstruction has been proposed as an alternative and more effective method in patients with obesity or esophageal dysmotility [31, 32]. In addition to this, resection of the distal esophagus, cardia and proximal stomach may be indicated if symptoms of the patient include dysphagia and pain. From a theoretical point, preservation of the duodenal passage by performing a Merendino procedure could reduce post gastrectomy symptoms as compared to RNY, but the data presented here are not supportive of this.

Previous studies of the Merendino reconstruction after resection for early Barrett cancer have shown varying short- and long-term results. In one of the largest series, Stein et al. [20] reported less postoperative complications compared to Ivor-Lewis esophagectomy and excellent long-term outcome as measured by the GIQLI (gastrointestinal quality of life index) instrument. In a more recent publication, where the EORTC QoL questionnaires were used, both short-term and functional results were similar after a Merendino reconstruction as compared to Ivor-Lewis procedure [33]. On the other hand, proximal gastrectomy with jejunal interposition in patients with non-advanced gastric cancer, which is a procedure similar to the Merendino, seems to have a superior functional outcome compared to total gastrectomy with RNY reconstruction [23, 24]. Obviously the importance of objectively assessing long-term data also in corresponding patient cohorts is warranted to assess the clinical place of the Merendino procedure. It is therefore particularly important to try to understand our experience of the poor results of the Merendino reconstruction among patients with a failed fundoplication. The high frequency of postoperative complications was expected, since this is frequent after most reconstructive procedure in the gastrointestinal tract. Objective findings of anatomical abnormalities among the patients were not always obvious. This may suggest that a general underlying pathology of gastrointestinal motility may have been present. Manometry of the esophagus and the interposed jejunal segment was available in 4 patients and in one of these cases aperistalsis of the jejunal segment was recorded. Barium swallow was performed postoperatively in 6 patients and showed a prolonged emptying of the jejunal segment or reflux in 5 and 4 cases respectively. The underlying mechanism for the poor function of the jejunal segment regardless of objective findings is obscure, but it can be hypothesized that the partial fundoplication that was performed may have contributed to the distal obstruction. However, also one patient with normal manometry findings had symptoms so severe, that a redo to RNY was performed. Thus, routine diagnostic methods may be inefficient in detecting possible motor disturbances of the gastrointestinal tract in this group of patients.

Finally, limitation of this study is that investigations done on a small sample of the group. Only four patients were investigated with HRM postoperatively and six patients with esophageal barium swallow. QOL data preoperatively don’t exist for these 12 patients and postoperatively were obtained from 6 patients. As a result it might be difficult to interpret the severity of the symptoms at the follow up.

## Conclusions

The conclusion to be drawn from this case series is that the Merendino procedure is, until proven otherwise, unsuitable for patients who undergo redo surgery after previously failed re-fundoplications.

## Abbreviations

DSRS: Dumping Syndrome
GERD: Gastroesophageal Reflux Disease
GIQLI: Gastrointestinal Quality Of Life
GSRS: Gastrointestinal Symptom Rating Scale
HRM: High Resolution Manometry
PPI: Proton-Pump Inhibitors
QoL: Quality Of Life
Qolrad: Quality Of Life in Reflux And Dyspepsia
